# Supplementary figures and images for: Cytokinin and Metabolites Affect Rhizome Growth and Development in Kentucky Bluegrass (Poa pratensis)
Source: Biology (Basel). 2023 Aug 11;12(8):1120. doi: 10.3390/biology12081120 (PMC10452147; doi:10.3390/biology12081120)

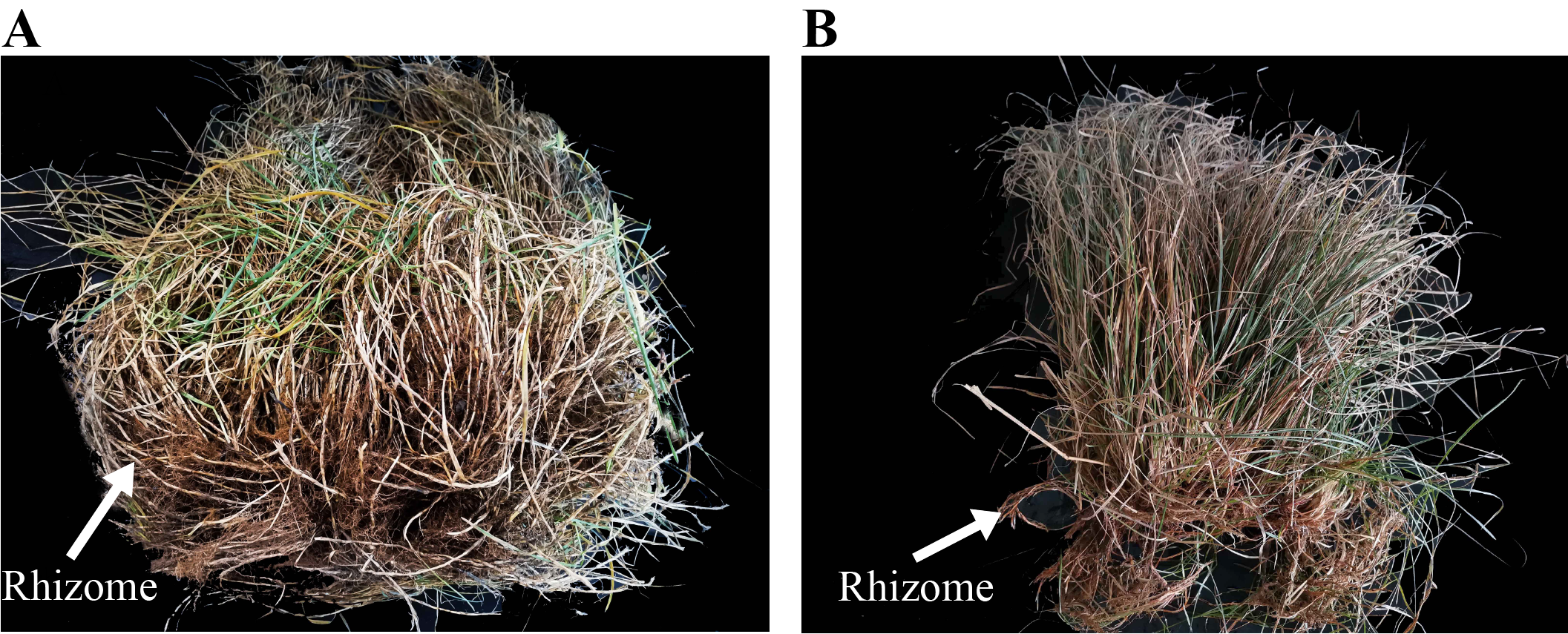

Supplement: Supplementary file 1 [file biology-12-01120-s001.zip › Figure S1.tif]

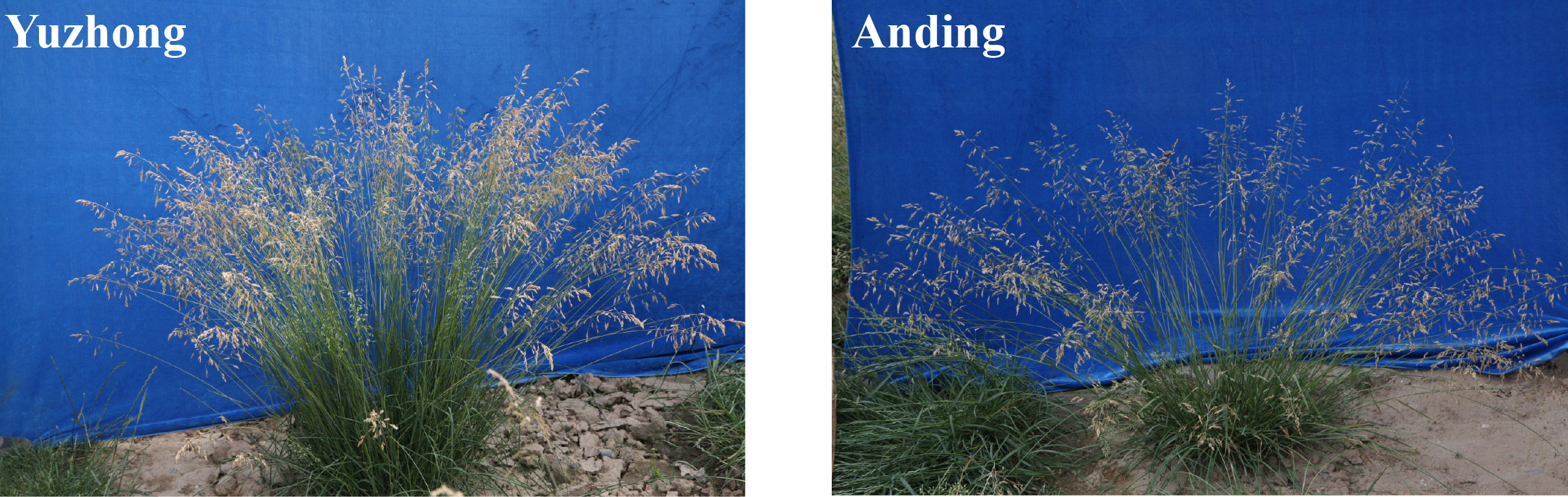

Supplement: Supplementary file 1 [file biology-12-01120-s001.zip › Figure S2.tif]
